# Recent Trends in Protective Textiles against Biological Threats: A Focus on Biological Warfare Agents

**DOI:** 10.3390/polym14081599

**Published:** 2022-04-14

**Authors:** Joana C. Antunes, Inês P. Moreira, Fernanda Gomes, Fernando Cunha, Mariana Henriques, Raúl Fangueiro

**Affiliations:** 1 Fibrenamics, Institute of Innovation on Fiber-based Materials and Composites, University of Minho, 4710-057 Guimarães, Portugal; ines.moreira@fibrenamics.com (I.P.M.); fernandocunha@det.uminho.pt (F.C.); rfangueiro@dem.uminho.pt (R.F.); 2 Centre for Textile Science and Technology (2C2T), University of Minho, 4710-057 Guimarães, Portugal; 3 CEB, Centre of Biological Engineering, LIBRO-Laboratório de Investigação em Biofilmes Rosário Oliveira, University of Minho, 4710-057 Braga, Portugal; fernandaisabel@ceb.uminho.pt (F.G.); mcrh@deb.uminho.pt (M.H.); 4 LABBELS—Associate Laboratory, 4710-057 Braga, Portugal

**Keywords:** advanced protection, protective textiles, biological warfare agents, antimicrobial, metal–organic frameworks, zinc oxide nanoparticles, chitosan-based nanoparticles

## Abstract

The rising threats to worldwide security (affecting the military, first responders, and civilians) urge us to develop efficient and versatile technological solutions to protect human beings. Soldiers, medical personnel, firefighters, and law enforcement officers should be adequately protected, so that their exposure to biological warfare agents (BWAs) is minimized, and infectious microorganisms cannot be spread so easily. Current bioprotective military garments include multilayered fabrics integrating activated carbon as a sorptive agent and a separate filtrating layer for passive protection. However, secondary contaminants emerge following their accumulation within the carbon filler. The clothing becomes too heavy and warm to wear, not breathable even, preventing the wearer from working for extended hours. Hence, a strong need exists to select and/or create selectively permeable layered fibrous structures with bioactive agents that offer an efficient filtering capability and biocidal skills, ensuring lightweightness, comfort, and multifunctionality. This review aims to showcase the main possibilities and trends of bioprotective textiles, focusing on metal–organic frameworks (MOFs), inorganic nanoparticles (e.g., ZnO-based), and organic players such as chitosan (CS)-based small-scale particles and plant-derived compounds as bioactive agents. The textile itself should be further evaluated as the foundation for the barrier effect and in terms of comfort. The outputs of a thorough, standardized characterization should dictate the best elements for each approach.

## 1. Biological Warfare Agents (BWAs)

In their daily lives, the world population is exposed to several threats that put their wellbeing and health at risk. Chemicals and BWAs are some of these threats [1]. BWAs include bacteria, viruses, fungi, and biological toxins and are responsible for several diseases such as anthrax, plague, tularemia, botulism, smallpox, and viral hemorrhagic fever [2,3]. BWAs are higher-risk agents for use as biological weapons and present variable mortality rates that depend on the biological agent and the mode of transmission/route of exposure. Their use for this purpose can promote large-scale morbidity and mortality, affecting a large number of people [2,4,5]. The early detection of a biological attack, namely of the agent involved, is crucial to their effective management and resolution, so that lower mortality rates can be attained. According to several criteria such as the ease of transmission, the severity of morbidity and mortality, and the probability of use, BWAs were classified by Centers for Disease Control and Prevention (CDCs) into different categories, specifically: Category A (highest risk to the public and national security—high priority agents); B (second-highest priority agents); and C (third-highest priority agents—emerging threats for disease) [6]. Some of the most relevant BWAs that are most likely to be used, with high mortality rates and a high potential for a major public health impact, belong to category A and are listed below.

### 1.1. Bacteria

#### 1.1.1. Anthrax

*Bacillus anthracis*, a spore-forming Gram-positive rod bacterium, is one of the most popular biological weapons in bioterrorism. It causes anthrax. A relevant example of a *B. anthracis*-driven biological attack happened in the 21st century (2001) in the US via the postal system (letters containing spores). This attack resulted in 22 infected people, of whom 5 died. *B. anthracis* is considered an effective BWA due to its ability to be aerosolized, form spores, and be easily cultured, as well as its capacity to remain viable for a long period of time in the environment. It can persist in the spore state for years or even decades, with the spores being extremely resistant to heat, irradiation, desiccation, and disinfectant action [7]. This bacterium is in the top list of the Category A priority pathogens [8]. *B. anthracis* has a short incubation period, usually 48 h, but it may be up to 7 days [9]. Its symptoms include fever, nausea, vomiting, sweats, dyspnea, respiratory failure, and hemodynamic collapse [10]. Toxin production (exotoxins: lethal toxin and edema toxin) is one of its virulence factors, along with the presence of a capsule that helps *B. anthracis* to evade host immunity. The natural incidence of anthrax is rare, occurring via contact with contaminated soil, infected animals, and infected or contaminated animal products [10,11,12]. The global anthrax prevalence is around 28%. The incidence was decreased during the 20th century. According to the World Health Organization (WHO), the estimated anthrax annual incidence is between 2000 to 20,000 cases [10,11]. The mortality rate is very high, mainly in cases of gastrointestinal anthrax, where the average is 25–60%, though it can reach 100%. Cutaneous anthrax, the most common form of disease manifestation, is known to provoke death in less than 20% of cases [13]. Injectional anthrax, a more recent form of the disease, has a mortality rate of 35% despite medical treatment [12]. The inhalational form has the worst prognosis, with a fatality rate of 80% or higher [14]. Prompt treatment with antibiotics is curative and enhances the chances of a full recovery [15]. Cutaneous anthrax is easily treated, while inhalational anthrax can be fatal even in cases of adequate treatment. Antibiotic resistance, a global concern, is evidenced by *B. anthracis* in its interaction with penicillin, highlighting the need for effective treatment options avoiding the use of this antibiotic, as well as of related β-lactam antibiotics. Nowadays, a combination of antimicrobials is used in the treatment of anthrax [16]. The multidrug regimen includes at least one bactericidal agent (such as ciprofloxacin or doxycycline) along with a protein-synthesis inhibitor (such as linezolid or clindamycin) to suppress toxin production. An antitoxin product (such as raxibacumab, anthrax immunoglobulin) is also recommended in parallel to the multidrug regimen to neutralize *B. anthracis* toxins by inhibiting the binding of protective antigens and the translocation of toxins into cells [17]. There are also vaccines available for anthrax, but only for people from 18 to 65 years old and at increased risk of exposure. Thus, the vaccine is recommended only for a minority of cases, namely professionals who come into contact with animal hides and fur, and some members of the army. Anthrax vaccine adsorbed (AVA) and anthrax vaccine precipitated (AVP) are licensed anthrax vaccines whose immunological component is the protective antigen, the major constituent of anthrax toxins [18]. Anthrax vaccines show a protective efficacy of 93% against inhalational and cutaneous disease [19].

#### 1.1.2. Plague

Another bacterium listed in Category A of bioterrorism agents is *Yersinia pestis*, a Gram-negative bacterium of the family Enterobacteriaceae that causes plague, famously known as “the Black Death”. It is associated with black scabs on skin sores. Although rare, plague caused by *Y. pestis* must be taken into consideration due to its possible intentional use as a bioterrorism weapon. The use of this biological agent as a biological weapon dates back to the Second World War [20,21]. Regardless, 75% of global plague cases have occurred in Madagascar, presenting an annual incidence of 200 to 700 suspected cases. Currently endemic, Madagascar endured an outbreak of plague in 2017, with a total of 2417 confirmed cases of plague and 209 patient deaths [22]. The mortality rates are indeed high, with pulmonary plague presenting a mortality rate of 40% and being fatal when untreated [23]. In parallel with *B. anthracis*, *Y. pestis* is one of the most virulent and deadliest BWAs, presenting mortality rates of 100% within 3 to 6 days postinfection [21,24,25]. *Y. pestis* is a nonmotile, non-spore-forming coccobacillus [20]. This bacterium has a short incubation period, usually 2 to 3 days, and symptoms include fever, headache, and general malaise. Plague can manifest in one of three clinical forms: bubonic plague, septicemic plague, and pulmonary plague, the latter being the most severe [26]. Plasminogen activator, Pla, is one virulence factor used by *Y. pestis* to overcome host immunity, since Pla adhesion and proteolytic ability have a crucial role in the manipulation of the fibrinolytic cascade and immune system [27]. Plague is a vector-borne illness transmitted by fleas from rodent reservoirs, but it can also be transmitted by direct contact or via aerosols (the inhalation of respiratory droplets). Fortunately, human cases are successfully treated with antibiotics (such as streptomycin, gentamicin, or ciprofloxacin). However, there are at least two cases of strains isolated in Madagascar (*Y. pestis* 16/95 and 17/95) exhibiting antibiotic resistance [20]. This poses an additional challenge for the control and management of the disease. Promising vaccine candidates are being created [28,29,30]. However, as of now, no licensed vaccine exists for plague. Once again, a quick diagnosis and treatment with antibiotics is crucial to a full recovery [23].

#### 1.1.3. Tularemia

Tularemia is another potential BWA [31,32]. In fact, tularemia is nowadays recognized as a reemerging disease due to the role of *Francisella tularensis* and its potential for misuse as a biological terrorism weapon [31,33]. This disease is caused by the Gram-negative coccobacillus-shaped bacterium *F. tularensis* [31,32]. *F. tularensis* is a pleomorphic, nonmotile and non-spore-forming bacterium. This infectious bacterium is easily disseminated by aerosols, has a low infectious dose, and is associated with rapid and fatal disease. Tularemia can be spread by vectors, direct contact with water contamination, sick animals, and inhalation. *F. tularensis* virulence factors consist mainly in their envelope (capsule, outer membrane, lipopolysaccharide, periplasm, inner membrane, among others), an outer structure that confers protection from host immunity and promotes infection and disease [34]. The incubation period for this bacterium is typically short, 3 to 5 days on average, up to 2 weeks [35]. Tularemia symptoms are highly variable and depend on the route of infection [36]. However, the most common include fever, headache, chills, malaise, and a sore throat [34,37]. The worldwide incidence of tularemia is not known [31], but it is known that the incidence of cases of tularemia declined during the 20th century [38]. Currently, in the US, about 200 cases of tularemia per year are reported [39]. Tularemia is a disease characterized by high morbidity and mortality. In untreated cases, the mortality rate ranges from 30 to 60%, while with treatment the death rate is less than 2% [40]. After tularemia recovery, some sequelae might occur, such as residual scars, lung and kidney damage, and muscle loss [36].

The treatment of this disease consists of antimicrobial therapy, specifically, antibiotics (quinolones, tetracyclines, or aminoglycosides) [32]. Although *F. tularensis* showed antibiotic resistance to, for example, ampicillin, meropenem, daptomycin, clindamycin, and linezolid, and is only susceptible to a small range of antibiotics, so far it has responded well to the antibiotics usually used to treat tularemia (gentamicin, ciprofloxacin, levofloxacin, and doxycycline) [41]. No vaccine is yet available for the prevention of this disease. However, clinical assays have been developed in order to find a vaccine against tularemia, and a mutant strain (∆pdpC) tested in animals (mice and monkeys) was demonstrated to be a good candidate for a live attenuated vaccine against *F. tularensis* [42].

### 1.2. Virus

#### 1.2.1. Smallpox

Although declared eradicated in 1980, smallpox, caused by the variola virus, remains a major threat to humanity due to its possible use as bioweapon [43,44]. The variola virus is an orthopox virus, one of the largest viruses to infect humans, belonging to the Poxviridae family [45]. It has a high mortality rate, high stability in an aerosol state, high transmissibility and high contagiousness among humans, a significant impact, and a great need for special preparedness. It is one of the most fatal diseases to have ever existed, presenting mortality rates of up to 30% (variola virus variant) [46]. The smallpox virus has a long incubation period, usually 11 to 14 days, and early symptoms include a fever and nonspecific macular rashes [47]. The variola virus is transmitted via respiratory droplets, cutaneous lesions, infected body fluids, and fomites. Smallpox sequelae include permanent scarring, which may be extensive; blindness resulting from corneal scarring; the loss of lip, nose, and ear tissue; arthritis; and osteomyelitis [48]. Smallpox inhibitor of complement enzymes (SPICE) and chemokine-binding protein type II (CKBP II) are considered two virulence factors of the variola virus, helping it evade the human immune system [49].

The smallpox vaccine, discovered by Edward Jenner in the 18th century, was the first vaccine to be successfully developed, involving the use of the cowpox virus to prevent smallpox [50]. In the 20th century, the first-generation vaccine comprised a strain of vaccinia virus followed by a second-generation vaccine based on the use of clones of the vaccinia viral strains used in the first-generation vaccine [51]. However, due to the controversial and severe adverse reactions to these vaccines, a safe and effective third-generation vaccine is being considered. KVAC103, a highly attenuated vaccinia virus strain, was recently proposed as such a candidate [45]. Tecovirimat, a small molecule used to treat smallpox, was the first smallpox antiviral therapeutic approved by the US Food and Drug Administration, but the smallpox virus has demonstrated resistance to it [44]. The latter constitutes a current concern that highlights the urgent need for multitherapeutic and effective strategies to fight this disease [52].

#### 1.2.2. Viral Hemorrhagic Fever

##### Ebola

The Ebola virus, which is suitable to be used as a BWA, belongs to the filoviridae family and is one of the causative agents of viral hemorrhagic fever in humans. This virus was first discovered in 1976 in the Democratic Republic of Congo, where the first Ebola outbreak occurred [53]. Currently, Ebola outbreaks continue to be recurrent in Africa, and its increased incidence requires an early detection in order to avoid the risk of an epidemic [54]. Since its discovery, over 20 outbreaks have occurred. Ebola fever is a fatal disease presenting a mortality rate ranging from 25 to 90%, and it is easily transmitted by direct contact with infected individuals (body fluids) [55]. The Ebola virus is a filamentous virus with a characteristic twisted thread shape. It has an incubation period of 2–21 days, with symptoms including fever, malaise, headache, diarrhea, and vomiting, and it can evolve into multiorgan failure (lungs, heart, kidney, liver), shock, and death [56]. Recovery is possible, though some sequelae can occur after disease recovery, including joint and vision problems, tiredness, and headaches [57]. The main virulence factors of the Ebola virus include some proteins such as virion proteins 35 and 24 (interferon antagonists) and glycoprotein, which interfere with the activation of a dysfunctional immune response and facilitate the attachment to host-cell surface receptor molecules and viral entry, respectively [58]. Currently, Ebola vaccines are being developed, including five promissory candidates, of which Ervebo, Zabdeno/Mvabea, and cAd3-EBOZ are the most advanced, based on a viral vector or on a modified version of a harmless surrogate virus. Among these, two are licensed (Ervebo and Zabdeno/Mvabea). The CanSino and GamEvac vaccines are also licensed, but only for emergency use in China and Russia, respectively. Although vaccines are available for Ebola, several questions remain unclear regarding their durability, safety, interaction with other therapeutics and vaccines, stability, etc. Other issues are related to vaccine costs, the narrow range of action (protection against only one species of Ebola virus), and the likely occurrence of intraspecies mutations that can affect the effectiveness of the vaccine [59]. Vaccinations are routinely administered for the Ebola disease only for individuals at high risk of exposure, due to the limited vaccine quantities, their unpredictable nature, and the relative rarity of Ebola outbreaks (mostly occurring in the regions of Central and West Africa) [60,61].

##### Lassa Fever

The Lassa virus is the causative agent of Lassa fever in humans, and it is an enveloped, single-stranded, bisegmented, negative-strand RNA virus belonging to the arenavirus family. It is responsible for 2 million cases of Lassa fever and 5000–10,000 deaths annually [62]. Lassa fever is an often-fatal hemorrhagic disease, first discovered in 1969 in Nigeria. This infection occurs mainly in West Africa and Nigeria and poses significant epidemic threats due to its high mortality (21–69% [63]) and morbidity rate and its highly contagious nature [64,65]. Lassa fever can have a zoonotic origin or can be transmitted by direct contact (aerosols or fluid secretions) with infected individuals. Its incubation period is 1 to 3 weeks. Lassa fever is normally asymptomatic in the initial stage or can present nonspecific symptoms such as fever, headache, malaise, and general fatigue, which can lead to a delay in diagnosis and treatment [66]. The progress of the disease leads to multiorgan collapse and hemorrhagic fever [67]. A prompt diagnosis and treatment is crucial to full recovery and, in fact, cases of severe Lassa fever with complete recovery were recently reported [66]. The recurrent outbreaks of Lassa fever and the emergence of the Lassa virus as well as its epidemic potential have highlighted the need for research into vaccines and treatments. To date, no approved vaccine is available to prevent the disease, and the therapeutic choices are limited [67]. Ribavarin, a synthetic nucleoside, is the only antiviral option available for the treatment of Lassa fever [68,69,70]. Currently, other therapeutic strategies are being developed and evaluated in humans and animal models. Of these, favipiravir and a human monoclonal antibody cocktail (Inmazeb) have shown potential to be used in clinical settings [62]. In parallel, several vaccine candidates are being examined, the most promising of which is based on the recombinant vesicular stomatitis virus, reassortants expressing Lassa virus antigens, and a deoxyribonucleic acid platform [71]; however, to date, no vaccine has passed the preclinical stage and evidenced both safety and efficacy in humans [62,71,72]. The main target used for the design of antibody-based therapeutics and Lassa virus vaccines is the envelope glycoprotein complex. This protein displayed on the surface of the Lassa virus can be considered a virulence factor, since it is essential for the attachment and entry of the virus into human cells [73].

### 1.3. Toxins

#### Botulism

In the case of botulism, another concerning BWA, the causative agent is the highly potent biological toxin botulinum neurotoxin produced by neurotoxigenic clostridia such as *Clostridium botulinum*. This toxin is the main virulence factor of this bacterium [74].

*C. botulinum* is a Gram-positive bacillus, spore-forming, anaerobic bacterium [75]. Natural cases of botulism are rare. Still, this toxin is easily produced, stored, and disseminated and presents extreme toxicity (lethal dose (LD50) = 1–3 ng/kg of body mass [76]). As a bioweapon, botulinum neurotoxin could be spread in food sources and via aerosolization. Between 1920 and 2014, only 197 outbreaks were reported, of which 55% occurred in the US, with an average of 110 cases reported annually. Botulism is a serious paralytic disease [77]. The toxin acts by blocking the release of a neurotransmitter, acetylcholine, at the neuromuscular junction, interfering with the nervous impulse and causing muscle paralysis [78].

Symptoms usually appear within 12–72 h after contact with the toxin. If untreated, botulism can progress to cause paralysis in various parts of the body, including respiratory muscles, leading to patient death. Patients with botulism may have a slow recovery that lasts days or even years. A prompt diagnosis and treatment can lead to full recovery in 2 weeks. In fact, reduced mortality was observed with the early administration of antitoxins and high-quality supportive care [79]. However, some sequelae can occur, such as feeling tired, shortness of breath, and ongoing breathing problems for a long time. Antitoxin therapy is the first-line therapeutic strategy used to promote toxin neutralization and elimination from blood circulation, being more effective when administered early in the course of the disease. It consists of antibodies or antibody antigen-binding fragments, whose purpose is to block the neurotoxin produced by *C. botulinum* [80]. However, patients may additionally require mechanical ventilation and/or other supportive measures until total recovery from paralysis. The availability of antitoxins and improvements in supportive and intensive respiratory care have substantially reduced the mortality rate by up to 5–10% in humans [77,81,82]. Unfortunately, although current treatment modalities can help to mitigate the progression/symptoms and accelerate recovery, no true antidote exists following exposure to botulinum neurotoxin [76,77,83]. Fortunately, vaccines are being developed to confer appropriate immune responses following incubation with the BWA, either in the case of a biothreat emergency or infectious disease outbreak [84,85,86].

## 2. COVID-19

COVID-19 is an acute respiratory illness that ranks third in terms of fatal coronavirus diseases threatening public health, with this kind of virus having emerged as a threat to people in the 21st century [87,88]. COVID-19 is caused by SARS-CoV-2, a beta-coronavirus, which was first reported in 2019 in China. Since then, SARS-CoV-2 has quickly spread all over the world, resulting in a pandemic situation that was declared by the WHO as a Public Health Emergency of International Concern. Its high morbidity and mortality rate have resulted so far in over 120 million infections and 2.5 million deaths worldwide in 1 year [89,90]. Although it has not been classified as a BWA by CDCs, and the origin/cause of its emergence is controversial, it is considered a global threat to health and safety and is already regarded as the greatest threat of this century [87]. The extremely high transmission rate of SARS-CoV-2 was one of the factors that contributed to its rapid propagation [91,92]. The virus is primarily transmitted by respiratory droplets and aerosol and contact routes. The implementation of the use of face masks or coverings was one of the strategies used to prevent virus transmission during the pandemic [90]. Such biological threats, whether of natural or intentional origin, highlight the extreme importance of bioprotective materials as fundamental to minimizing the consequences of this kind of threat.

## 3. Antimicrobial Activity Test Methods

Microorganisms can be carried by textiles and even multiply themselves in this environment, which is the reason why this kind of substrate is regarded as a possible vector of infection and disease transmission in hospitals and communities [93]. On the other hand, textiles can be used as means of protection against the transmission of diseases, including biological and chemical threats. In reality, there is a growing body of research concerning the development and application of textiles for military use, aiming at providing protection in a wide range of hostile environments and with a rapid effect on bacteria, fungi, viruses, and even toxins.

Biological threats do not have simulants in the same way as CWAs; however, for bacteria, several standard strains are typically used to evaluate the biocidal capacity of proposed textiles [94]. These selected strains are easily handled in the laboratory using well-established assaying protocols and representative bacterial strains of each group, including *Staphylococcus aureus* (Gram-positive bacteria) and *Escherichia coli* (Gram-negative bacteria) [94,95,96,97,98]. Gram-positive and Gram-negative bacteria differ in their cell wall structure, and this difference affects their susceptibility to antimicrobials [99,100]. The cytosol of Gram-positive bacteria is encircled by a cytoplasmic membrane attached to a thick peptidoglycan layer, while the cell wall of Gram-negative bacteria contains two distinct lipid membranes, the cytoplasmic cell membrane and the outer membrane, with a thin layer of peptidoglycans in between [100,101,102]. In addition to the two aforementioned microorganisms, which are the most commonly used in this type of evaluation, *Candida albicans*, a unicellular fungus, is another regularly assessed species [103]. However, many others are also routinely used, of which *Pseudomonas aeruginosa* and *Klebsiella pneumoniae*, both Gram-negative bacteria, can be emphasized [95,103,104,105,106,107,108,109]. The viricidal potential is often examined using model viruses such as bacteriophage MS2 (a surrogate of the SARS-CoV-2 virus) and P22 (a surrogate of the *Salmonella* virus), even though rotavirus and severe-acute-respiratory-syndrome-associated coronavirus (SARS-CoV) have been used to test potentially protective textiles [94]. In short, test microorganisms should be selected according to the intended application of the textile [99].

Test standards for antimicrobial textiles usually consist of two types of testing method: qualitative (first-step screening of the antimicrobial activity of antimicrobial textiles) and quantitative [110]. Among the various standards available, AATCC 147, JIS L1902, AATCC 100, and ISO 20645 are the most relevant examples [100,110]. Qualitative methods (agar diffusion assay) are based on the measurement of the halo, a clear zone of inhibition around the sample. In quantitative methods, the evaluation of the antimicrobial activity is more efficient and is based on the measurement of the number of microorganisms (or colony-forming units) after 18–24 h of contact with the textile material [93]. The different standards differ in the inoculation method, sample size, inoculum concentration, culture medium, and buffer formulation, among other things [110].

## 4. Biological Protective Textiles

**Figure 1:**
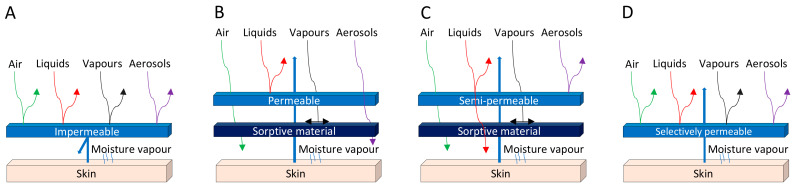
Schematic representation of the different types of conventional biological protection: (**A**) impermeable membrane; (**B**) air-permeable shell layer; (**C**) semipermeable shell layer; (**D**) selectively permeable membrane (adapted from [111,112]).

The development of protective clothing is crucial nowadays, as there are increased levels of harmful biological threats, both for military forces and civilians [111]. The main purpose of barrier textiles is to protect the user against external hazards such as BWAs while maintaining safety and comfort next to the skin [112]. Figure 1 illustrates, in a simple manner, the different types of conventional biological protection, namely an impermeable membrane (A), an air-permeable shell layer (B), a semipermeable shell layer (C), and a selectively permeable membrane (D). However, most of the available protective clothing systems rely on passive protection, acting as a full barrier against air, vapors, and liquids, as in hazardous materials (HAZMAT) suits (Figure 1A) [111]. Materials that are chemically or mechanically unresponsive to the environment must be engineered to meet performance specifications under worst-case-scenario conditions, often sacrificing performance for the sake of other parameters [113]. Air-permeable overgarments are most frequently composed of an activated-carbon layer to adsorb toxic vapors, designed to be worn over battledress duty uniforms (Figure 1B) [111,112]. Although activated-carbon adsorption material has protective properties, it is limited by a nonselective adsorption, poor protection performance against large toxic liquid droplets, and secondary pollution. Hence, current needs, new materials, and new technologies are acting together to promote the advances of permeable protective suits in pursuit of high performance, multifunctionality, lightweightness, and comfort [114]. The development of new protective clothing with different features that can adsorb hazardous agents is envisioned, which can be accomplished by using different fibrous materials and by following a specific design. Selectively permeable fabrics are important to improving the user’s comfort by reducing the airflow through the fabric layers while keeping a high water-vapor permeability [114]. As an example, the integration of electrospun nanofiber membranes in textile fibrous structures produces a high aerosol filtration efficiency, good air permeability, low surface density, and low-pressure loss, thanks to the small but highly interconnected pores and large surface area of built nanofibers [1,114]. In addition, active protection appeared as a promising concept to detect and inactivate/degrade microorganisms and BWAs, while considering that materials capable of responding to their environment may achieve optimal performance under a much wider set of conditions [1,113]. This can be achieved either by using fibers such as the ones prepared via electrospinning or by functionalizing textiles with nanomaterials that possess those capabilities.

The development of biological protective clothing depends on a combination of different requirements, such as a barrier to liquids, water vapor permeability, and stretch properties. However, it also depends on parameters such as weight and comfort for the wearer, which will ultimately influence the level and durability of the protection. The type of biological threat also impacts this selection and constitutes one of the reasons why the requirements must be established beforehand [115].

This section will focus on the different materials and techniques, from the conventional to the innovative protection methods.

### 4.1. Fibrous Materials

Protective clothing can be achieved through the usage of several different fibrous materials, which are listed in this subsection with regard to the current solutions and the new developments.

#### 4.1.1. Conventional Protection

Commonly used materials for totally impermeable protective clothing are butyl and halogenated butyl rubber, neoprene, and other elastomers [115]. Even though they are effective in conferring a barrier against liquids, vapors, and aerosols, they impede moisture vapor from travelling from the user’s body and skin to the environment. This is why fibrous materials are exploited in the development of protective clothing.

Conventionally, synthetic fibers such as polyester, polyethylene, polypropylene, polyamide, and polyurethane are used to fabricate protective clothing [116]. Natural fibers such as cotton, wool, and those regenerated from naturally available polymers can also be employed to provide not only protection (mostly thermal) but also comfort. These are advantageous for protective textiles in comparison to synthetic fibers due to their biocompatibility and low cost, among other things, but they normally require combination with high-performance fibers or post-treatment and finishing processes [1,117,118,119]. While collecting data on protective textiles, the dominance of cotton fiber is evident. This is mainly because of its natural comfort, appearance, and excellent performance, such as its alkali resistance, hydrophilicity, and moisture retention. Cotton fiber has, however, poor crease recovery, poor dye fixation, microbial growth, photo-yellowing, and poor color fastness properties that need to be improved [120]. Nevertheless, numerous strategies are being developed to overcome such limitations.

#### 4.1.2. Innovative Protection

Some specific fibers can be used in a way that provides sensing and responsive capabilities, making active protection possible. For instance, high performance fibers such as ceramic fibers, carbon fibers, stainless steel, and aluminum fibers can be employed [121]. However, most of these lack moisture management properties and are not durable, which is the reason why they are normally mixed with conventional fibers or interwoven in fabrics.

### 4.2. Fibrous Structures

Different fibrous structures can be developed, and these are presented in this subsection with respect to conventional and active innovative protection.

#### 4.2.1. Conventional Protection

Completely impermeable suits can be achieved by film-laminated fabrics as a full hazardous barrier (Figure 1A). However, these do not meet the comfort requirements after a long operational time, as the water vapor permeability is high, which causes heat stress for the wearer.

Air-permeable fabrics are usually made of a woven shell fabric, an activated-carbon layer, and a liner fabric (Figure 1B) [115]. The activated-carbon layer is crucial for adsorbing toxic chemical vapors, since the outer layer is permeable not only to air, liquids, and aerosols, but also to vapors.

**Figure 2:**
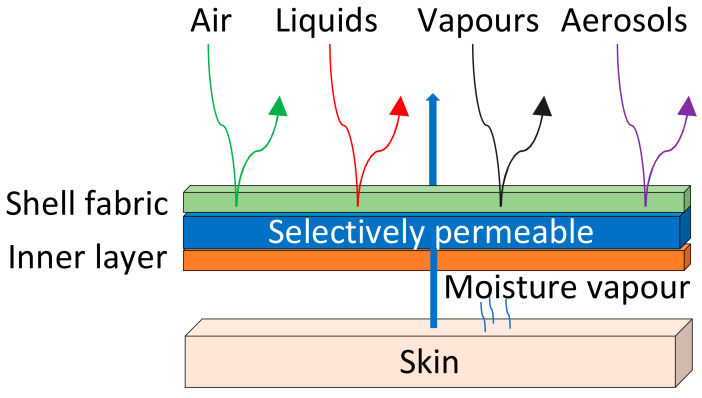
Detailed schematic drawing of a selectively permeable membrane (adapted from [111,112]).

Another technique to improve the comfort of protective clothing is to use an impermeable material as a barrier for the outer part and a more breathable material for the inner part. To this end, semipermeable fabrics are designed (Figure 1C). In addition, a perm-selective membrane that allows the permeation of water vapor molecules but inhibits the passage of larger organic molecules (Figure 1D) can also be developed. Several materials, mostly polymers, have been used for these semipermeable or selectively permeable membranes (SPMs), such as poly(vinyl alcohol), cellulose acetate, cellulosic cotton, or poly(allylamine). The development of different membranes for protective textiles has been thoroughly reviewed, from the barrier films and breathing membranes to the future directions that advocate the use of selectively permeable barriers, which are schematically represented in Figure 2.

Nonwoven fabrics made of a three-layered composite (spun-bonded, melt-blown, spun-bonded) are also a common option for biological protection [1,122]. However, the passage of BWAs through multilayered protective clothing is rather complex and thus must be thoroughly studied. The combination of different layers and barrier properties, in addition to the skin breathability and comfort, must be optimized. The gas/vapor transport by diffusion and convection should be studied and correlated with the vapor and liquid sorption of the protective fabrics in order to assess the degree of protection. The results highly depend on the properties of the materials used, such as yarns and fibers, but also on the fabric construction and clothing assembly [123]. Additionally, the intertwined interactions between some parameters are key, such as the fabric thickness, adsorption, and air permeability properties. Modeling work appears as a promising tool for the prediction and representation of air flow through designed fibrous arrangements and structures. With this, 2D structures can be developed and assessed in terms of their performance.

#### 4.2.2. Innovative Protection

There is an increasing interest in the development of active solutions for protection, with the ability to neutralize BWAs. Smart textiles have appeared and present a wide range of applications, including self-cleaning, phase-transition fabrics and protective clothing [124,125]. Nanotechnology has appeared as a promising solution to develop protective textiles with specific functionalities, such as UV protection, antimicrobial activity, and chemical resistance [112]. Particularly, the use of nanotechnology in chemical, biological, radiological, and nuclear (CBRN) protection clothing has arisen as an excellent possibility. The properties of nanoparticles, nanowires, nanotubes, nanostructures, and nanocomposites are distinctive from those of bulk materials.

An ultrahigh surface area and high surface concentrations are desired for the attachment of biocides and the destruction of adsorbents. This way, nanofibrous networks and consequent closely packed assembly has turned electrospinning into a highly attractive technique to produce membranes for biological protection [126]. A matt of nanofibers can be deposited, creating a randomly oriented fibrous assembly comparable to a nonwoven fabric, but this random assembly can also be collected and oriented into a yarn. The production of electrospun nanofiber-based membranes is promising for the achievement of a clothing system with a lighter weight. In addition, the small pores between fibers improve particulate retention, absorbing hazardous microorganisms. Electrospun polyurethane fibers have been shown to be effective in regard to their elasticity. Since biological agents penetrate fabric and skin in a slow manner, the decontamination of the surface is crucial and does not require immediate neutralization to make sure that the fabric and skin are not penetrated [111]. This, once again, points to the functionalization of fabrics as a promising solution. The combination of this with structured multilayered protective clothing can be highly advantageous for future developments.

### 4.3. Bioactive Agents

The latest research has directed its efforts at the study of metal–organic frameworks (MOFs); quantum dots; and inorganic particles integrating silver (Ag), copper (Cu), zinc (Zn), and titanium (Ti) cations. Glimpses of the potential of natural polymer chitosan (CS) or derivatives as BWA-counteracting agents, applied as a coating layer or in the form of organic particles (loaded or not with plant-derived compounds such as plant extracts and essential oils (EOs)) can be perceived. Hydrogen-bonded organic frameworks (HOFs), which emerged recently, are also showing high potential to act as self-cleaning materials. The following sections will describe the aforementioned bioactive agents, unveiling the details of their biocidal potential, mechanisms of action, and known limitations.

#### 4.3.1. Metal Organic Frameworks (MOFs)

Zr is ubiquitous in nature, favoring research with Zr-based porous materials, namely zirconium dioxide (ZrO_2_) or zirconia, which have outstanding optical and electrical features for the development of transparent optical devices, capacitors, fuel cells, and catalysts. Recently, a new class of Zr-based highly porous hybrid materials has emerged, consisting of inorganic metal-ion or metal-oxide clusters bridged by organic linkers, possessing tunable pore sizes, surface area, pore volumes, and responsiveness to visible light [127]. Zr-based MOFs are attracting tremendous attention from the scientific community and have started to become known for having the ability to degrade BWAs (research was first directed at CWAs) and thus having great potential as protective layers in suits or masks or in air purification systems (capturing toxic gases), since the metal-containing secondary building units function as Lewis acid sites for the catalytic hydrolysis of hazardous compounds [57,127,128,129].

The overall use of MOFs is, however, hindered by the intractable powdery or crystalline forms of the prepared catalysts, which additionally require complex instrumental settings for their processing [128,129]. Another limitation stems from the fact that, in order for them to act as antimicrobial agents, their structure needs to be robust; a release of metal ions (or active linkers) leads to the collapse of the structure. As a consequence, these structures may only be used as temporary microbicidal surfaces. Regardless, MOFs have been instrumental as light-induced disinfectants for pathogens [94]. Scarce, but solid, literature exists linking MOFs to military biological protection. Cheung and colleagues [128] screened an MOF derivative against both CWAs and BWAs. They introduced regenerable MOFs, using a N–chlorine biocide, into a textile via a porous UiO-66-NH_2_ (a stable zirconium-based MOF with -NH_2_ functional groups in its organic ligands) as the regenerable carrier. The active chlorine atoms were bonded to the amine-functionalized linker in the ordered framework to form chloramine groups by a simple immersion process in commercial bleaching solutions. The active-chlorine-loaded MOF/fiber composite (UiO-66-NH-Cl/PET; PET = polyethylene terephthalate) quickly killed both Gram-positive *S. aureus* and Gram-negative *E. coli* bacteria, as well as the SARS-CoV-2 virus, after a few minutes. The active N−Cl in the modified Zr-MOF coating was stable and regenerable, acting through the slow release of active chlorine through the pores of the MOF when in contact with the pathogens. The active chlorine could then be generated after water (from the surroundings) was used to hydrolyze the N−Cl to form HClO. The porosity of the MOF allowed the diffusion and slow release of the active chlorine, as the chlorine on the surface was consumed. N–chloramides had previously been deemed as self-decontaminating and regenerable against multiple CWAs, with the goal of using them within military textiles [130].

#### 4.3.2. Inorganic NPs

Nanoparticulate systems are colloidal-sized particles with diameters between 1 and 1000 nm. Their size offers a high surface/volume ratio and a correlation with the structural sizes of biological components: they are small enough to pass through biological barriers, internalize some target cells, and influence multiple cellular processes [131]. Inorganic NPs comprehend metallic, bimetallic, metal oxide, and magnetic elements in their structure [132], with metal oxide NPs containing silver (Ag), copper (Cu), zinc (Zn), or titanium (Ti) cations being the most studied in the fight against microbes such as those that constitute BWAs, alone or combined for synergistic activities. However, there are different types of inorganic NPs with particular characteristics and mechanisms of action against pathogens. In addition to their inherent physical structure, one of the main antimicrobial mechanisms exerted by inorganic NPs is reactive oxygen species (ROS) production [133]. When in direct contact with cells, NPs act through electrostatic attraction, ligand–receptor interactions, hydrophobic reactions, and van der Waals forces. Bioactive metallic ions are likewise released through the metal oxides that absorb the cell’s peripheral layers, allowing them to interact with the functional groups of biomolecules, such as proteins and nucleic acids, extra- or intracellularly. This triggers cell metabolic and structural changes, generating homeostatic imbalances [134].

Silver (Ag) NPs wield bacteriocidal effects on both Gram-negative and Gram-positive bacteria at relatively low drug dosages, but side effects such as cytotoxicity in vitro and allergic responses in vivo may happen in the case of an overdose or prolonged use. Moreover, Ag NPs are prone to aggregate and have poor stability, even though stabilizers such as polyvinylpyrrolidone (PVP) or sodium dodecyl sulphate can be used to assist in shielding the corona of Ag NPs from disintegration, augmenting their diffusivity and contact with the microbes while decreasing their toxicity. Alternatively, Ag NPs can be anchored to the surface of some materials, thus relieving the weight of Ag NP-associated disadvantages [135]. In all cases, the main mechanism of action of Ag NPs against pathogens require the attachment and interaction of multiple NPs to the cell surface [53]. This induces the disruption of the microbe outer layer functions and the dissipation of the proton motive force. Small Ag NPs of a few nanometers may even alter the morphology of the cell wall, increasing their internalization and ultimately killing the cell [102]. Compared to Ag and gold (Au), copper (Cu) is cheaper and more attainable, biocompatible, and environmentally friendly. Cu NPs dissolve faster than other noble metals by outward ion release. Cu is an essential element to life, and it is a key regulator in several pathways that are essential for living. As such, Cu ion release can take part in some of these pathways. On the other hand, Cu NPs may accumulate in the body or release too many ions, causing long-term toxicity or contributing to the development of related diseases [134]. The work of Bhattacharjee et al. [104] disclosed that the application of either Ag NPs or Cu NPs enhanced the antimicrobial potency of the built structures for future use in protective clothing and medical textiles. Ag and Cu have broad intrinsic spectra of antimicrobial activity. The first biological barrier of microorganisms is traditionally negatively charged. Hence, these cationic NPs are able to disrupt cell membranes due to electrostatic attraction and form hydroxyl free radicals, resulting in lipid and protein oxidation. The results of the antibacterial activity underlined Ag NP-embedded samples as the most efficient bactericides. A plausible explanation could be the formation of an oxide layer on the Cu NPs, given that Cu NPs are highly susceptible to oxidation, when stored under ambient conditions. However, considering the toxicity of Ag NPs and the much lower cost of Cu, Cu NPs are becoming more attractive nowadays.

ZnO NPs are well-known for their low cost, availability, biocompatibility, biodegradability, and hexagonal prism shape, which allows an increase in surface roughness that ultimately enhances cell anchorage points. Their UV protection, photocatalytic activity, antimicrobial, self-cleaning, energy-harvesting, and biosafety features can confer multiple functionalities to their substrates: water resistance, antimicrobial action, UV blocking, flame retardancy, corrosion inhibition, and electrical conductivity [136]. Zn-doped NPs are indeed capable of endowing a fabric (e.g., cotton-derived) with superhydrophobic properties that facilitate cleaning [136], among other functionalities, including a microbicidal capacity [137]. Noorian and colleagues [138] showed excellent UV protection and significant antibacterial efficacy even after 20 washing cycles and 100 abrasion cycles following the in situ production of ZnO NPs, showcasing their potential for use in advanced protective textiles. The suggested mechanisms of action were again ROS formation, Zn-ion release, membrane dysfunction, and NP internalization, as taken from the literature. Nonmetal and metal doping may effectively change the active wavelength threshold of the absorbed light to the visible area [139], thus enhancing the antimicrobial characteristics in settings where UV light is absent. Doping metals such as Ag, Cu, Au, La, Sm, and Fe and nonmetals such as N, F, C, and S on the ZnO structure [139], or even carbon-based materials [140], enables the possibility of achieving such outcome. However, problems related to the stability, dispersion, and crystalline structure control of ZnO NPs in an aqueous medium seriously hinder the industrial application of this bioactive agent [141]. Moreover, although ZnO NPs offer significant safety and biocompatibility, several authors argue that their toxicity within biological systems should be better understood and controlled [142,143,144]. These toxic effects have so far been attributed to the high solubility of the particles, resulting in the cytotoxicity, oxidative stress, and mitochondrial dysfunction of mammalian cells [144].

Finally, the work on TiO_2_ NPs has revealed good photochemical and chemical stability, hydrophobicity, biocompatibility, a low cost, and high photocatalytic and hydrophilic activity. These NPs are activated under UV-light irradiation and generate electron–hole pairs that dispense Ti^4+^ to Ti^3+^ cations and oxidize O^2−^ anions to oxygen atoms. The ejection of oxygen atoms from the TiO_2_ complexes produces oxygen vacancies that are occupied by water molecules, which in turn leave OH groups on the surface of TiO_2_ NPs and make them hydrophilic. The generated electron–hole pairs induce bacterial growth inhibition and produce ROS. The addition of carbon-based materials such as graphite enlarges the activation range of TiO_2_ nanoparticles to visible light and causes increased hydrophilic, photocatalytic, and antibacterial properties. To stabilize TiO_2_ and TiO_2_ composites, they can also be uniformly dispersed in polymeric substrates [145]. Görgülüer et al. [146] revealed that the photocatalytic activity of TiO_2_ NPs was improved by the deposition of metal NPs (notably Ag NPs) on the TiO_2_ surface, since the formation of a Schottky barrier at the metal–semiconductor interface resulted in the more efficient capture of photogenerated electron–hole pairs. Moreover, the surface plasmon absorption of Ag NPs can broaden the absorption spectrum in the visible region. Regardless, a lotus leaf effect on the tested assemblies [147] and antimicrobial activity [148,149] are generally present when TiO_2_ NPs are added to the proposed substrates. However, their toxicity to human health and the ecosystem is also a considerable concern related to their extended use [150].

#### 4.3.3. Organic Small-Scale Particles

Organic small-scale particles comprise polymeric structures that are widely studied in the literature as drug delivery systems. Specifically, proteins, lipids, polysaccharides, nucleic acids, and other biomolecules are capable of being processed into small-scale particles, with increasingly significant research pinpointing their utility for drug delivery. These biomolecules can also be combined with inorganic nanomaterials to produce hybrid materials showcasing features from both types of material [151].

Some recent studies have explored CS-based small-scale particles loaded with plant-derived molecules to prevent or control infections while interspersed within fabrics to function as protective textiles. CS is widely recognized for its tuneable biocompatibility, bioactivity, chemical versatility, and ease of processing into a variety of structures, thus finding itself considered of high value for numerous applications [95,131,152,153,154,155,156]. Plant extracts or essential oils (widely used as folk medicine) are increasingly being studied as antimicrobial agents, as several natural drugs have already been approved for clinical use. Their modes of action comprise: the inhibition of cell wall synthesis, the permeabilization and disintegration of microbial peripheral layers, the restriction of microbial physiology, oxygen uptake and oxidative phosphorylation, efflux pump inhibition, the modulation of antibiotic susceptibility, biofilm inhibition, the hindrance of the microbial protein adhesion to the host’s polysaccharide receptors, and the attenuation of pathogen virulence [131]. EOs in particular act through their inherent hydrophobicity, which enables them to accumulate in the cell membrane, disturbing its structure and functionality and causing an increase in their permeability to a point at which cell lysis and death is unavoidable [153]. Notwithstanding, their loading onto/into organic particles has also been the object of several studies, as a way of enhancing molecules’ biostability and bioactivity, along with controlled release, thus holding the power to provide strong and durable effects. CS-based small-scale organic carriers have tremendous potential [131,153,156,157,158]. Recent efforts from the team of Bouaziz et al. [96] demonstrated that coacervated CS microcapsules, with cinnamon EO in their cores, could substantially inhibit the growth of the tested Gram-positive and Gram-negative bacterial strains. The antibacterial results were mainly due to the cinnamaldehyde (the major constituent of their cinnamon EO batch) after the oil release from the microcapsules and were not attributed to the CS itself or to the built architecture, even though the authors did not test unloaded particles. However, a fact is that the antimicrobial potency of CS alone is highly variable, depending on its cationic nature, when its amine groups are protonated (which traditionally occurs at 9.5 < pH < 6.5, depending on the degree of acetylation). CS either accumulates at the cell surface, forming a polymer layer that prevents substance exchanges such as nutrient intake and metabolic disposal, or, as in the case of CS with a low Mw, reaches the intracellular compartments, adsorbing electronegative substances, disrupting the cells’ equilibrium, and killing them [131]. If the environmental pH is above CS’s pKa, the inhibitory effect is instead governed by hydrophobic interactions and the chelating capacity of divalent metal ions rather than the electrostatic interactions between its protonated amines and anionic bacterial outer-layer structures [153]. The known limitations are associated with batch-to-batch variability, stability in physiologically compatible media, burst release, washing durability, and poor mechanical properties [131].

Another study, defended by Wang et al. [159], created hydrogen-bonded organic frameworks (HOFs), which are supramolecular self-assembled π-conjugated structures of rigid and large functional tectons that demonstrated a significant enhancement in daylight-driven ROS generation capacity and ROS storage lifetime under dark conditions. After daylight stimulation for 2.5 min, the fluorinated HOF-101-F/fiber killed almost 95% of *E. coli*. The composite shows excellent sterilization efficiency under light irradiation and dark treatments for five cycles without decreasing its performance. HOF-101-F, after exposure to daylight for 30 min, could kill over 99.99% of *S. aureus*, *Klebsiella pneumoniae,* and *Mycobacterium marinum*. However, the applicability of HOFs is still in its infancy, with large conformationally flexible building blocks remaining a challenge because of rigid molecule approximations and limitations in the accuracy of force fields to rank diverse energy landscapes reliably, especially those where interpenetration is present [160].

Regardless, one truth is that the presence and efficiency of organic NPs within textiles is still sporadic.

#### 4.3.4. Carbon Nanodots

Carbon dots can be divided into carbon nanodots (CNDs), carbon quantum dots (CQDs), and graphene quantum dots (GQDs) [151]. CQDs are newly emerging quasi-spherical NPs with a particle size of 1–10 nm. These carbon-based materials have high temperature resistance, outstanding electrical/thermal conductivity, high plasticity, corrosion resistance, UV blocking, a high adsorption rate, good water solubility, excellent biocompatibility, low toxicity, and a high catalytic performance [107,161,162]. Some studies can be found describing their potential use as fluorescent probes against BWAs, in particular dipicolinic acid, a biomarker of *B. anthracis* [162,163] or of *E. coli* [162], as a result of their low environmental hazard, high selectivity, greater sensitivity, good biocompatibility, changeable fluorescent properties, and excitation-dependent multicolor emission behaviour. CQDs are composed of sp2 carbon atoms formed in planes, with each carbon atom being mainly connected to the three nearest neighbors with a distance of 120 degrees. The implantation of oxygen-, sulfur-, and nitrogen-containing functional groups can be introduced to the sides of graphite sheets to overcome the intersheet van der Waals forces that subsequently result in the enlargement of the interlayered spacing. However, despite this, the applicability of CQDs is still narrowly exploited in batteries, fuel cells, supercapacitors, and transistors, with sensing and bioimaging being indeed more actively explored [162]. One particular study reported the integration of carbon quantum dots clustered from the fluorescent aromatic compound named 4–(2,4–dichlorophenyl)–6–oxo–2–thioxohexahydropyrimidine–5–carbonitrile within a textile matrix for military protective garments [107]. CQDs were able to completely eradicate all the tested species: *S. aureus*, *E. coli*, and *C. albicans*. Even after 10 washing cycles, microbial inhibitions were substantially high. CQDs, specifically their lateral functional moieties, act by creating microbial oxidative stress intracellularly under visible light and in an aqueous medium. Oxidative stress can be defined as differences in the subcellular and tissue compartmentalization of ROS that contribute to stress responses, provoking altered cellular activities, cell proliferation, extracellular matrix synthesis, the production of matrix-degrading enzymes, and cell apoptosis. ROS comprise singlet oxygen, singlet sulfur, singlet nitrogen, and hydroxyl free radicals. In lethal doses, ROS directly guide nucleic acids to fragmentation; corrupt gene expression and protein synthesis values; incite lipid peroxidation, gradual cell wall destruction, and necrosis/apoptosis; and encourage microbial cell death [107,164]. Regardless, high toxicity due to the use of heavy metals in production, complex processing methodologies, and poor control over dot size have been related to this type of bioactive agent [165,166].

#### 4.3.5. Graphene and Derivatives

Graphene is a thick layer of sp2-hybridized carbon atoms arranged in a honeycomb-like crystal lattice [167]. Graphene has become one of the most studied carbon-based materials in recent years due to its excellent mechanical characteristics, high electrical conductivity properties, high Joule-heating capacity, high UV shielding, rapid heat dissipation, high hydrophobicity, high thermal stability, high antimicrobial activity, and high biocompatibility. However, it is limited by low fabrication rates and a high cost, in addition to a strong aggregation tendency and hydrophobicity, which leads to insolubility in aqueous media [104,168,169,170]. Consequently, graphene derivatives such as graphene oxide (GO) and reduced GO (rGO) have been produced. GO can be synthesized from graphite powder. It has several oxygen-containing functional groups, which turn it into a chemically versatile material. However, in some cases, these oxygen-based functional groups reduce its functionality. Thus, it is reduced using chemical, electrochemical, or thermal approaches, creating rGO. rGO shows properties similar to pristine graphene and also relatively good conductivity. It can be easily prepared in the desired amounts from cost-effective GO [151]. GO and rGO are able to form covalent or hydrogen bonds with textiles such as cotton or silk via their carboxyl, hydroxyl, and epoxide groups. The addition of rGO to cotton or silk, as shown by Bhattacharjee et al. [104], resulted in mild antibacterial activity, which seemed to derive from the scissoring action of its sharp creases/edges and the generation of oxidative stress in the pathogenic cells through electron transfer. rGO has been shown to react with lipids, DNA, and amino acids via electrostatic and π−π stacking interactions. The reaction between rGO’s oxygen and the cell wall polysaccharides of bacteria has also been reported [104,168]. However, it remains difficult to precisely control the compositions and sizes of graphene sheets, which heavily affects the performance of the derivatives [171].

**Figure 3:**
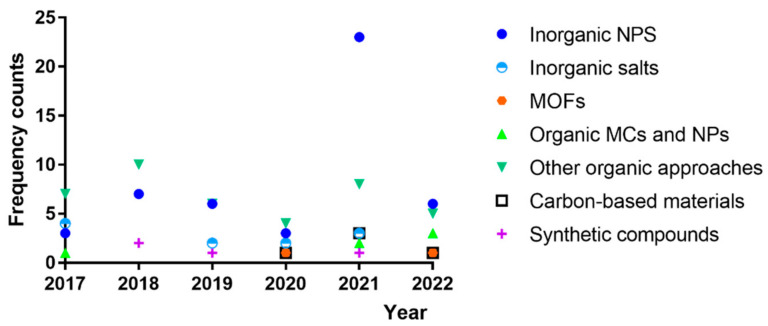
Approximate frequency counts of the usage of different categories of antimicrobial agents within protective textiles (intended for the military, first responders, or civilians) within published literature of the last 5 years (database: Scopus).

Figure 3 illustrates the current trends in the use of different types of antimicrobial agents within protective textiles, for military use or otherwise. It presents approximate frequency counts of the use of these materials within the published literature of the last 5 years (database: Scopus). Inorganic NPs are the major contributors to these numbers, but natural approaches fall shortly behind. Of the former, Ag-based strategies are the most commonly explored. On the other hand, research using natural biocidal approaches is highly unfocused, even though many studies explore plant extracts, CS and derivatives, CS-based architectures, and plant-extract-loaded CS-based small-scale particles. However, it becomes clear that the quest to find suitable bioactive agents has been narrowing over the years, with bioactive agents such as ZnO or CuO NPs gaining more importance lately. Besides, MOFs as well as carbon-based materials such as CQDs and graphene derivatives emerged around 3 years ago for this type of application. In addition, natural approaches have appeared, emphasizing the potential of CS, particularly if processed in the form of small-scale particles carrying biomolecules such as plant-derived compounds.

### 4.4. Textile Fabric Functionalization Methods

Textiles can carry microorganisms and also promote their survival, proliferation, and endurance. When a fabric is used for clothing, an infestation may create infections and constitute a biological threat. Antimicrobial functional finishes are therefore applied to textiles to protect the wearer and the fabric itself [172]. Various techniques exist to immobilize bioactive agents onto textile fibers, each one carrying its specifications, advantages, and limitations, with the fabric being previously treated and functionalized in order to improve the impregnation of the selected bioactive agents, as well as their durability within the textile. The dip-pad-cure method, the dip-and-dry method, the exhaustion method, the spray-dry method, the spray-cure method, the pad-batch method, and sol-gel and sonochemical coatings are a few relevant examples of the impregnation methods of bioactive agents [105,173]. However, coating and laminating procedures are increasingly important techniques for adding value to textiles, including coating approaches such as the lick-roll method; direct coating (knife on air, knife over table, knife over roller, knife over rubber blanket); foam coating; foam and crushed-foam coating; transfer coating; kiss-roll coating; rotary-screen printing; spray coating; calendar coating; hot-melt extrusion coating; and rotogravure [174].

Starting with MOFs, recent antimicrobial stars, some interesting studies have been performed. The work of Cheung and colleagues [128] stands out, as PET textiles had UiO-66-NH_2_ MOFs grown in situ following chlorination with a hypochlorite bleach solution to obtain regenerable N–chlorine MOFs coating the textile. The same occurred elsewhere [106], but this time ZIF(Ni), ZIF-8(Zn), and ZIF-67(Co) were the MOFs synthesized into cotton fabrics. A silicate modification acted as a crosslinker between cotton on one side and ZIF-MOFs on the other, thereby increasing the number of MOFs adsorbed onto the fabrics. The fabrics were scoured for dirt removal or even bleached for discoloration [106,128], and sometimes functionalized to gain functional dopamine moieties [175] or the previously mentioned silicate modification [106] to reinforce binding with the bioactive agents through covalent bridges.

The same trend has been observed with inorganic NPs, with most of the NPs being grown in situ following textile incubation with metallic precursors. Despite their well-known handicaps, Ag NPs continue to be the most studied inorganic NPs in protective textiles, although often in combination with other microbicidal enhancers. Textile functionalization with the bioactive agents occurs mostly via the in situ formation of NPs [95,104,105,176,177]. As an example, El-Naggar and colleagues [105] showed that bleached and mercerized (an alkaline treatment to improve affinity towards subsequent chemical modifications) cotton fabric was rendered more hydrophilic through plasma treatment, then washed with a nonionic detergent to remove impurities and silanized to encourage metal–ligand binding with the Ag NPs. Silanization treatment forms silane groups that act as fiber–NP coupling agents, creating a siloxane bridge between the two components [178]. Finally, the treated fabric was immersed in a solution carrying metallic precursors, sonicated, padded, squeezed, and cured for thermal reduction to form Ag NPs. Görgülüer et al. [146] washed rayon fabric in an acetic acid solution and in a wet surfactant so that any chemical finishing, such as silicon, and softening on the fabric could be efficiently removed. Afterwards, the fabric was immersed in TiO_2_ NPs; poly(dimethylsiloxane) (PDMS) to functionalize the later NPs with hydrophobic moieties; AgNO_3_ and NaBH_4_ as metallic precursor and reducing agent, respectively; and finally, tetrahydrofuran (THF) to assist in the production of compact and spherical Ag NPs. Samples were ready for characterization following a drying step. While using ZnO NPs to guarantee bacterial cell death in desized and bleached cotton fabrics, Noorian et al. [138] also washed the fabric in nonionic detergent, before performing oxidization by periodate and treatment with 4-aminobenzoic acid ligands (PABA). NPs were similarly built in situ after the immersion of the fabric in a ZnO precursor, ultrasonication, and chemical reduction.

The integration of CQDs into cotton fabric that had been scoured, bleached, and cationized with 3–chloro–2–hydroxypropyl trimethyl ammonium chloride (C_6_H_15_Cl_2_NO), was indeed very simple [107]; it was achieved by dissolving previously prepared CQDs, impregnating the fabric with them while stirring, and drying. The addition of an rGO coating through a dip-dry process onto fabrics composed of cotton or silk [104] that had been previously washed with acetone and hot water and functionalized with a silane derivative allowed increased quantities of Ag and Cu NPs to be added subsequent to the composition, particularly with cotton, which is richer in hydroxyl groups than silk.

Botelho and team [95] washed PA taffeta and submitted it to plasma treatment. CS was then added through the dip-dry method, followed by the already prepared Ag NPs. Dip-pad-dry was the immobilization technique also selected by Verma et al. [120] to integrate dissolved CS, along with citric acid (C₆H₈O₇) to act as a linker to the enzymatically desized and scoured cotton fabric, with sodium hypophosphite (NaPO_2_H_2_) as the catalyst; this worked as a mordant to enhance the dyeability of the cotton. Samples were then padded, dried, and cured. A final step included a dyeing process with onion-skin dye. Some studies have additionally integrated plant-derived molecules into/onto CS-based small-scale particles [96,131,179]. Singh et al. [179] used the emulsification of gelatin and rosemary EO followed by ionic gelation between gelatin and CS to encapsulate the EO and produce a stable shell. Linen fabric was dipped in a microcapsule (MC) dispersion and low-temperature curable acrylic binder, padded, and dried. Verma and colleagues [96] encapsulated cinnamon EO within CS MCs produced by simple complexation with Tween 20. Dense taffeta cotton fabrics, which had been desized, bleached, and mercerized, were dipped into an MC dispersion and a binding agent (dimethyloldihydroxyethylene urea, DMDHEU), padded, dried, and cured; they were then autoclaved and stored. In another study, Wang and colleagues [159] explored HOFs that carried building units incorporating CH_3_-, F-, or NH_2_-groups on the ortho-position of the phenyl ring of the benzoic acid and were produced via a sol-gel method. These were spray-coated onto woven and knitted cotton fabric, as well as commercial chirurgical disposable face masks; dried; washed in acetone to remove unbound agent and solvent; and then dried again.

As mentioned above, multiple bioactive agents have been tested with textiles, alone or combined in order to obtain synergistic effects in the fight against pathogens. Many authors are also aware of the need to obtain durable bioactive effects, namely by retaining the bioactive compounds attached to fibers [128,146,159]. It is, however, noticeable that the past two years, during the COVID-19 pandemic situation, have been key for attempts to control the washing durability of finished fabrics, thereby responding to a major concern of the textile finishing industry [96]. Some authors have even followed standardized protocols to assess such features (the KS K ISO 6330 [104], IS: 3361-979 [120], AATCC-61 [180], or AATCC 2010 [107] standards), thus proving that the required bioactivity is present even after laundering activity. Table 1 summarizes the main, and representative, antimicrobial protective textiles designed for military purposes or for general use.

## 5. Conclusions

The SARS-CoV-2 pandemic, which has generated a global health and economic crisis, has shown us that we need to be better prepared for the next global threat, which may be caused by pollutants, chemical toxins, or biohazards [94,182]. The urgency of obtaining effective solutions to degrade BWAs such as anthrax [7,8] has been increasing in response to a recent risk increment associated with the possible use of biological weapons. Consequently, it is essential to develop personally protective systems that can actively protect their user, ideally without compromising his/her comfort, which is highly pertinent, for instance, while working in war zones for long periods of time [1,183]. Active protection is preferred when compared to passive protection, since it allows the total degradation of hazards and does not require a post-decontamination process [1]. We need to develop protective textiles in which infectious pathogens cannot survive, proliferate, and persevere so easily [94]. The damage inflicted by these harmful agents can be avoided by taking appropriate preventive measures [184]. The development of active fibrous structures with MOFs, inorganic agents (e.g., ZnO NPs), carbon-based materials (such as CQDs and graphene or its derivatives), and/or organic players such as chitosan (CS)-based layers or small-scale particles (loaded or not with plant-derived compounds) as bioactive agents is paving the way in the manufacture of protective textiles such as army suits, general protective clothing, or face masks that can efficiently counteract the survival of these pathogens. The decision as to the best bioactive agents strongly depends on the specific application and requirements, but the advantage of inorganic NPs seems clear. The research studies presented and interlinked here reinforce that ZnO NPs are one of the most promising materials for the development of high-performance textile products and should therefore be intensively investigated in the future, as is also argued elsewhere [185]. Strategies should be applied to counteract their current limitations. Bioactive features should be thoroughly examined and controlled via standardized protocols.

The addition of such elements into selectively permeable barrier textiles would fill a gap that currently exists, for instance, in charcoal-based protective suits that are designed to solely confer passive protection, and it would likely not add significant extra weight to the composition [1,115,186,187]. Aspects such as fabric composition and construction and clothing assembly should be paid more attention to, as they can substantially contribute to the required barrier effect and comfort. Moreover, charcoal-based protective suits and similar items, such as the majority of face masks that are currently employed, are limited to a single use. Hence, contemporary challenges include the development of circular and multifunctional protective textiles with durable effects, regenerable bioactive agents, and recyclable/degradable materials [188,189]. The use of natural compounds can be a great strategy and an excellent alternative to the use of synthetic ones, due to their high abundance in nature, low cost, and biodegradability. The use of simple and greener methods is also preferred [1]. Overall, this area is presently a hot topic in both the scientific and industrial communities, being an object of intense research, yet it is unfortunately still highly dispersed. It thus seems to be imperative to apply all the efforts to successfully innovate and create scientific and technological breakthroughs, while rigorously defining all the requirements for a fully functional protective textile, performing all the needed standardized protocols to adequately evaluate each hypothesis, and allowing the results to speak for themselves regarding the definition of the best elements and/or combinations to use, so that substantial improvements in the field of antimicrobial protective textiles (namely against BWAs) can be achieved. On the verge of contact with dangerous pathogens, we seek products that actually work, making this entire pursuit worthwhile.

## Figures and Tables

**Table 1:** Recent trends (2020–2022) in antimicrobial protective textiles designed for military purposes or for general use.

Fabric		Bioactive Agent	Impregnation Method	AM Testing				Protective Textile	Ref.
Details	Cleaning and/or Pretreatment			Cell	Method	Main Results	Durability		
Woven and knitted cotton fabric, plus commercial chirurgical disposable face masks	-	HOF-101-R (R=H, CH_3_, F, NH_2_), obtained by sol-gel method	Spray coating: HOF-101 tecton derivatives (1 mg/mL in DMF) were sprayed on various fiber materials (1 × 1 cm^2^) for 10 s and dried (100 °C, 1 h). The procedure was repeated enough times until the sprayer was empty. Fibers were washed by acetone 3 times and dried (100 °C, 1 h).	*S. aureus*, *E. coli*, *K. pneumoniae,* and *M. marinum*	Shake-flask method, under simulated daylight and dark conditions	After illumination under simulated daylight for 2.5 min, the HOF-101-F/fiber killed 95% of *E. coli*. Following 12 h of solar irradiation and exposure to bacteria for 2 h, cell death was ≈46%. Performance maintained after light irradiation and dark treatments for 5 cycles. Over 99.99% of bacteria was eliminated after daylight treatment for 30 min. Antibacterial performance under complete dark conditions without preirradiation was much slower.	Washed in water without observable HOF loss.	Face masks	[159]
PET	Scoured in 3% NaOH solution (90 °C, 20 min), then washed with water	Regenerable N-chlorine, loaded into Zr-MOF UiO-66-NH_2_	In situ MOF synthesis: PET textile (20 cm × 20 cm), BDC-NH_2_ (90 mmol, 16.2 g) and ZrOCl_2_·8H_2_O (60 mmol, 19.4 g) mixed in water (400 mL) and TFA (200 mL) in a sealed 1 L Schott bottle, sonicated for 0.5 h, placed at 100 °C for 6 h, cooled to RT, washed by water (2 × 500 mL) and acetone (3 × 500 mL), dried at RT, and activated at 110 °C for 24 h under dynamic vacuum.	*S. aureus*, *E. coli*, and SARS-CoV-2	Modified AATCC 100–2004 (with textile “sandwiched” using another identical sample for full contact), SEM of harvested bacteria, anti-SARS-CoV-2 virus test	Bacteria: 7-log reduction within 5 min. SARS-CoV-2: 5-log reduction within 15 min.	23% loss in chlorine content after 40 days storage, sealed, under ambient conditions, still enabling total sterilization.	Cloth against BWAs and CWAs	[128]
100% plain-woven cotton, 185 gm/ m^2^	Scoured, bleached, then cationized with C_6_H_15_Cl_2_NO (50 °C, 2 h)	CQDs clustered from synthesized TM	Dip-dry: 0.25 g of prepared components (TM or CQDs) dissolved in 25 mL of CHCl_3_. Fabric (0.25 g) impregnated in 0.25 g of TM or CQDs (1 h, continuous stirring), then air-dried.	*S. aureus*, *E. coli*, and *C. albicans*	Kirby–Bauer disk diffusion technique, MIC determination	82%, 71%, and 62% growth inhibition, respectively, in 24 h.	68%, 63%, and 67% growth inhibition, respectively, after 10 washing cycles.	Military clothing	[107]
Pristine CNWs fabricated from pulp and lyocell fibers	Drying (90 °C, 5 h) and hydrofobization with CI, plus UV-induced grafting of PTB	PHMG or NEO	Outer layer: grafting of antiviral/antibacterial agents by the ring-opening reaction of the PTB with -NH_2_ of PHMG or NEO onto hydrophobic CI-functionalized CNWs. Middle layer: the same onto pristine CNWs.	*S. aureus*, *E. coli*, HcoV-229E virus, and SARS-CoV-2 virus	Colony count method and antiviral testing	Bacteria: >99.99%, 99.99 ± 0.01% growth inhibition rate after 10 min of incubation with CNWs-PTB-PHMG. Sars-Cov-2: 16.23 ± 1.69% survival after ~0.1 min with CNWs-PTB-NEO, 99.84% ± 0.14% after 30 min with CNWs-PTB-PHMG.	-	Face masks	[181]
100% plain-weave cotton fabric: 80 ends/inch, 75 picks/inch, and 168 (g/m^2^)	Scoured, bleached, and C_8_H_11_NO_2_-modified (immersion in C_8_H_11_NO_2_.HCl solution at pH 8.5, 24 h)	Ag NPs	Dip-dry: immersion in 10 mM AgNO_3_ (continuous stirring, 30 °C, 8 h) and vacuum-drying (12 h, 40 °C).	*S. aureus* and *E. coli*	ASTM E2149-01	Bacterial reduction of 86% for *S. aureus* and 93% for *E. coli* following 1 h of incubation, 100% after 24 h.	~98% bacterial reduction after 20 washes.	Functional textiles	[177]
Woven viscose (120 g/m^2^)	Fabric phosphorylation: immersion in DAPH at a molar ratio of 1:1; urea was also included as 3 equiv of DAHP, then rinse with water	ZPT	Dip-pad-dry: padding with 0.5 wt % aqueous solution of N_2_O_6_Zn·6H_2_O via the 2-dip-2-nip method. Then, water-soluble NaZPT was added at a molar ratio of 1:2 with respect to the metal precursor. Immersion in a ZPT ligand solution (2 h, 40 °C, orbital shaking at 120 rpm). Drying (80 °C, 10 min), curing (150 °C, 2 min), and rinsing with water.	*S. aureus*, *E. coli*, and *C. albicans*	Qualitative Kirby−Bauer disk diffusion method; quantitative AATCC-100, OD600, and bacteria survival (CFU) measurement methods; SEM and quantitative antifungal assay	Viscose-ZPT induced high ZoI (48 or 53 mm, respectively, against *S. aureus* or *E. coli*).	Viscose-ZPT induced high ZoI after 20 washes (38 or 43 mm, respectively, against *S. aureus* or *E. coli*). 96–97% growth inhibition (20 washes).	Protective clothing	[180]
100% cotton or silk	Acetone and hot water (60 °C) washed; air-dried; soaking in coupling-agent solution (pH 4–5, C_9_H_20_O_5_Si:water = 1:15) for 4 h at 60 °C; air-dried	rGO and Ag/Cu NPs	Immersion in 0.25 mg/mL rGO suspension (RT, 4 h), air drying (3 times), separately soaked in 0.05 M AgNO_3_ and CuSO_4_·5H_2_O solutions (2 h), air-dried, immersion in 2% wt/V Na_2_S_2_O_4_ solution (chemical reduction, 4 h, 80 °C, 100 rpm), washed in water, dried (hotplate at 60 °C), and heat-treated in a vacuum oven (20 min, 175 °C).	*S. aureus*, *E. coli*, *P. aeruginosa*, and *C. albicans*	CFU counts	69–99% (*S. aureus*), 92–100% (*E. coli*), and 97–100% (*P. aeruginosa*) growth inhibition, especially with Ag NPs after 24 h; 63–69% *C. albicans* growth inhibition with Cu NPs (50% with Ag NPs), namely using cotton.	85−99% growth inhibition against Gram-negative bacteria; 62 to 90% against *S. aureus* after 10 washing cycles.	Protective clothing	[104]
Woven cotton fabric (areal mass density: 280 g/m^2^; threads/cm: warp 48 ± 2; weft 37 ± 1; and CIE whiteness 80)	Desized, bleached, and mercerized	CS MCs, prepared by simple emulsion (with Tween 20) and loaded with cinnamon bark EO	Immersed in MCs (80 g/L) and the binding agent (40 g/L, DMDHEU), padded (wet pick up of 80%), dried (90 °C, 15 min), cured (150 °C, 5 min), autoclave-sterilized, and stored at RT.	*S. aureus* and *E. coli*	Diffusion assay method	90% (*S. aureus*) and 97% (*E. coli*) growth inhibition.	69% MC remaining after 5 washes, 12.5% after 10 washes.	Protective textiles	[96]
100% cotton knitted fabric (194 g/m^2^) with (1 x 1) interlock structure	Cleaned with acetone and water, mercerized	Ag NPs	Immersed into a solution of C_6_H_8_O_6_ (5 min), dried (5 min, 80 °C); immersed into AgNO_3_ solution (5 min), dried (5 min, 80 °C); 1–3 cycles. Encapsulation in a silicone binder solution in acetone at a ratio of 1:7 for 5 min (1 time), dried (10 min, 80 °C).	*S. aureus* and *E. coli*	AATCC 147, agar diffusion assay	Higher ZoI for 1-cycle samples after 24 h (0.531 mm with *S. aureus*, 0.25 mm with *E. coli*).	-	Protective textiles	[176]
Woven cotton fabric	Enzymatic desizing and scouring	CS and onion-skin dye	Dip-pad-dry: dip within CS (4%), C₆H₈O₇ (6%), and NaH_2_PO_2_ (5%) at 1:30 material:liquor ratio (pH 5, 90 °C, 45 min), pad (P = 2 kg/cm, expression of 70–75%), dry (100 °C, 5 min), and cure (140 °C, 4 min). Dyeing with onion-skin dye (exhaustion method): 6% dye, pH 5.5, 90 °C, 75 min, 1:30 material:liquor ratio.	*S. aureus* and *E. coli*	AATCC Test Method100, shake-flask	*S. aureus* (98.03%) and *E. coli* (97.20%) growth reduction after 24 h.	Reduction in *S. aureus* growth from 96.84 to 80.14% and *E. Coli* from 93.20 to 80.74% after 5–20 washing cycles.	Protective textiles	[120]
Rayon fabric	Acetic acid (3 g/L) and TEGO^®^ wet surfactant (2 g/L) (Evonik) solution in DW (pH 3.5, 20 min), oven-drying	TiO_2,_ Ag NPs	Dip-dry: immersion in coating mixture (60 mL of 5% TiO_2_ NPs + 9.7 mL PDMS + 8 mL of 1 M AgNO_3_ + 10 mL 0.017 M NaBH_4_ + 30 mL THF) 10 min, drying (70 °C, 4 h).	*S. aureus* and *E. coli*	Agar diffusion assay	ZoI of 14.44 mm (*S. aureus*) and 13.12 mm (*E. coli*) after 24 h.	Water contact angle remained nearly constant (152.3°) after 20 laundering cycles.	Multifunctional textiles	[146]
Polyamide taffeta (52 warp and 32 weft yarns, 100 g/m^2^)	Washing, plasma treatment (RT, atmospheric pressure, width of 50 cm, gap distance of 3 mm, 10 kV, 40 Hz, 5 times, both sides)	Ag NPs, CS	Dip-dry: dip in each solution (5 min, RT) and dry (50 °C, 20 min).	*S. aureus* and *P. aeruginosa*	ASTM-E2149-01, shake-flask	*S. aureus* (80%) and *P. aeruginosa* (60%) growth reduction after 2 h.	-	Face masks	[95]
Bleached and mercerized cotton fabric	O_2_ plasma treatment (13.56 MHz, 3 min, 400 W, 200 cm^3^/min, 0.003 mbar); washing with nonionic detergent (C₃₂H₆₆O₉, 10 mmol); sonication (30 min); air-drying and washing with water; dipping in acetone solution of C_9_H_22_O_3_SSi (1%, 24 h); curing (75 °C, 30 min); rinsing with water	Ag NPs	In situ synthesis of Ag NPs: dip in 0.1–4 wt % CH_3_AgNO_2_, sonication (15 min), padding, squeezing, and curing (130 °C, 5 min).	*S. aureus*, *E. coli,* and *C. albicans*	Agar diffusion assay	Clear and large ZoI after 24–48 h.	-	Multifunctional textiles	[105]
Plain cotton fabric (135 g/m^2^)	Immersion in 4 mg/mL C_8_H_11_NO_2_. HCl (pH 8.5)	ZIF-8	Immersion in Zn(NO_3_)_2_.6H_2_O (0.893 g, 15 mL) solution + C_4_H_6_N_2_ (0.985 g, 15 mL) solution, autoclaving (100 °C, 12 h), washing, and drying (60 °C).	*E. coli*	Disc diffusion method	Defined ZoI after 24 h.	-	Multifunctional textiles	[175]
Cotton fabrics (shibeka, honeycomb, and crepe)	Bleached	CS or Ag NPs	Dip-dry: immersion in CS solution (10 min), squeezing for 100% wet pickup (constant pressure), drying (80 °C, 4 min), and curing (140 °C, 2 min); immersion in Ag NP dispersion (100–300 ppm), squeezing for 100% wet pickup (constant pressure), drying (80 °C, 3 min), and curing (140 °C, 2 min).	*S. aureus*, *P. aeruginosa, C. albicans,* and *A. niger*	Disc diffusion method	20 or 13 (*S. aureus*), 15 or 11 (*P. aeruginosa*), 13 or 21 (*C. albicans*), and 12 or 11 mm (*A. niger*) with 6% CS (Crepe) or 300 ppm Ag NPs (Shebika), respectively, after 24 h.	-	Protective textiles	[108]
Desized and bleached cotton fabric (100% cellulose, 117.5 g/m^2^)	Washed (30 min, 50 °C, nonionic detergent Adrasil HP P-836, 1 g/L, 1:60 L:G), water-rinsed, dried at RT; periodate oxidation in phosphate buffer (pH 8, L:G 1:50, dark), addition of NaIO_4_ (5 g/L, 30 min, ultrasonication at 20 kHz, 750 W at 70% efficiency), water-washed, dried at RT; PABA treatment (10 g/L, 2 h) using acetate buffer solution (pH 5.5, ultrasonication), water-washed, dried at RT	ZnO NPs	In situ synthesis of ZnO NPs: immersion in 1 mM ZnCl_2_ solution (30 min) and ultrasonication (pH 10 for 30 min by adding 4 g/L NaOH). Ultrasonication (extra 30 min, 60 °C), water washing, and drying (120 min, 110 °C).	*S. aureus* and *E. coli*	AATCC 100-2004, 24 h	99.9% (*S. aureus*) and 99.4% (*E. coli*) growth inhibition.	93.7% or 95.3% (*S. aureus*) and 93.4% or 95.4% (*E. coli*) after abrasion or washing process, respectively.	Protective textiles	[138]
Scoured and bleached plain-woven 100% cotton fabrics (165 gm/m^2^)	Silicate modification: immersion in 100 mL of 5% NaOH (50 °C, 5 h, stirring), addition of 6 mL C_3_H_5_ClO (5 h reaction), water and anhydrous ethanol washing, drying (60 °C); silicate mixture synthesized by dropwise addition of SiC_8_H_20_O_4_ (12 mL) and methanol (80 mL) to a flask with 30 mL of ammonia and 320 mL of methanol; stirring 3 h, curing (110 °C, 1 h)	ZIF(Ni), ZIF-8(Zn), and ZIF- 67(Co) MOFs	In situ synthesis of MOFs: immersion, separately, in 50 mL of methanol with metal salts (0.736 g of Ni(NO_3_)_2_, 0.758 g of Zn(NO₃)₂, and 0.733 g of Co(NO_3_)_2_), stirring 1 h at RT; pour three solutions individually from C_4_H_6_N_2_ (1.623 g in 50 mL of methanol) above the three mixtures, stir 8 h; ethanol-wash and dried (vacuum, 60 °C, 12 h).	*S. aureus*, *B. cereus*, *E. coli*, and *C. albicans*	Kirby−Bauer disk diffusion method, overnight	ZoI: 25 (*S. aureus*), 23 (*B. cereus*), 15 (*E. coli*), 22 (*C. albicans*) for cotton–silicate–ZIF(Ni).	ZoI: 19 (*S. aureus*), 18 (*B. cereus*), 12 (*E. coli*), 18 (*C. albicans*) for cotton–silicate–ZIF(Ni) after 5 washing cycles.	Protective textiles	[106]
Inner layer: polystyrene fiber 3-ply twisted yarns (tex: 0.058, 0.115, or 0.230); outer layer: 3-ply twisted single yarns with PCMs, including use of functional fibers Resistex^®^ Silver	Washed with 2.5 g/L nonionic detergent Felosan RG-N, 2.0 g/L Na_2_CO_3_, 3.0 g/L water softener CalgonVR Power (60 °C, 60 min), rinsed with 1 g/L acetic acid solution, centrifuged, air-dried	Silver	None	*S. aureus*, *E. coli*, and *K. pneumoniae*	EN ISO 20645	Low bacterial growth.	-	Multifunctional socks	[109]

PET: poly(ethylene terephthalate);TFA: trifluoroacetic acid; RT: room temperature; AATCC (American Association of Textile Chemists and Colorists); TM: 4–(2,4–dichlorophenyl)–6–oxo–2–thioxohexahydropyrimidine–5–carbonitrile; MIC: minimum inhibitory concentration; CNWs: cellulose nonwovens; CI: cyclohexyl isocyanate; UV: ultraviolet; PTB: poly(thiiran-2-yl methyl methacrylate-2-(4-benzoyl phenoxy)ethyl methacrylate; PHMG: polyhexamethyleneguanidine; NEO: neomycin sulfate; DAPH: diammonium hydrogen phosphate; ZPT: zinc pyrithione; ZoI: zone of inhibition; CFU: colony-forming units; DMDHEU: dimethyloldihydroxyethylene urea; ZIF-8: zeolite imidazole skeleton-8; PABA: 4-aminobenzoic acid ligand; L:G: liquor-to-fabric ratio; PCMs: phase-change materials.
